# Microcystic Adenoma of the Pancreas: Observation or Treatment?

**DOI:** 10.1155/1993/63464

**Published:** 1993

**Authors:** T. E. Pavlidis, J. N. Thompson, R. C. N. Williamson

**Affiliations:** Hepatopancreatobiliary Unit Department of Surgery Hammersmith Hospital Du Cane Road London W12 ONN UK


*HPB Surgery*, 1993, Vol. 6, pp. 295–299
Reprints available directly from the publisher
Photocopying permitted by license only

© 1993 Harwood Academic Publishers GmbH
Printed in the United States of America

### MICROCYSTIC ADENOMA OF THE PANCREAS: OBSERVATION OR TREATMENT?

T.E. PAVLIDIS, J.N. THOMPSON and R.C.N. WILLIAMSON
*Hepatopancreatobiliary Unit, Department of Surgery, Hammersmith Hospital, Du Cane Road, London W12 0NN, UK*

*(Received 1 June 1992)*

KEY WORDS: Pancreatic cystadenoma, pancreatic resection, percutaneous biopsy

### INTRODUCTION

Cystic lesions of the pancreas include inflammatory cysts (pseudocysts), congenital cysts and neoplastic cysts. Pseudocysts, which predominate, are identified by having no epithelial lining. Neoplastic cysts represent only 10–15 percent of all pancreatic cysts but must be carefully differentiated from pseudocysts to ensure proper management[1–6]. Among the different types of cystic neoplasm (Table 1), microcystic adenoma is a benign variant. It is also called serous cystadenoma to distinguish it from the common mucinous cystadenoma, which has a malignant potential. We report two patients with serous cystadenoma of the pancreas, highlighting the use of percutaneous biopsy in its management.

### CASE REPORTS

### *Case No: 1*

An 85-year-old woman was referred for management of an upper abdominal mass of unknown origin. She had been complaining of lethargy and weight loss for a year but had no gastrointestinal or other specific symptoms. She had previously undergone a left nephrolithotomy and thyroidectomy and had been treated for pneumonia one year before presentation. On examination there was a hard,

Address correspondence to: Mr J.N. Thompson.

nodular, mobile and ballotable mass in the left hypochondrium approximately 8 × 4cm. She had a normocytic, normochromic anaemia (Hb 10.2 g/dl), a slightly elevated serum alkaline phosphatase (342 iu/l) and elevated plasma glucose (9.3 mmol/l). Thyroid function tests were normal.

Ultrasound examination of the abdomen confirmed an 8cm solid mass in the left upper quadrant. CT scan showed that this mass was situated in the lesser sac; it was related to the left lobe of liver and stomach but was of uncertain origin (Figure 1).

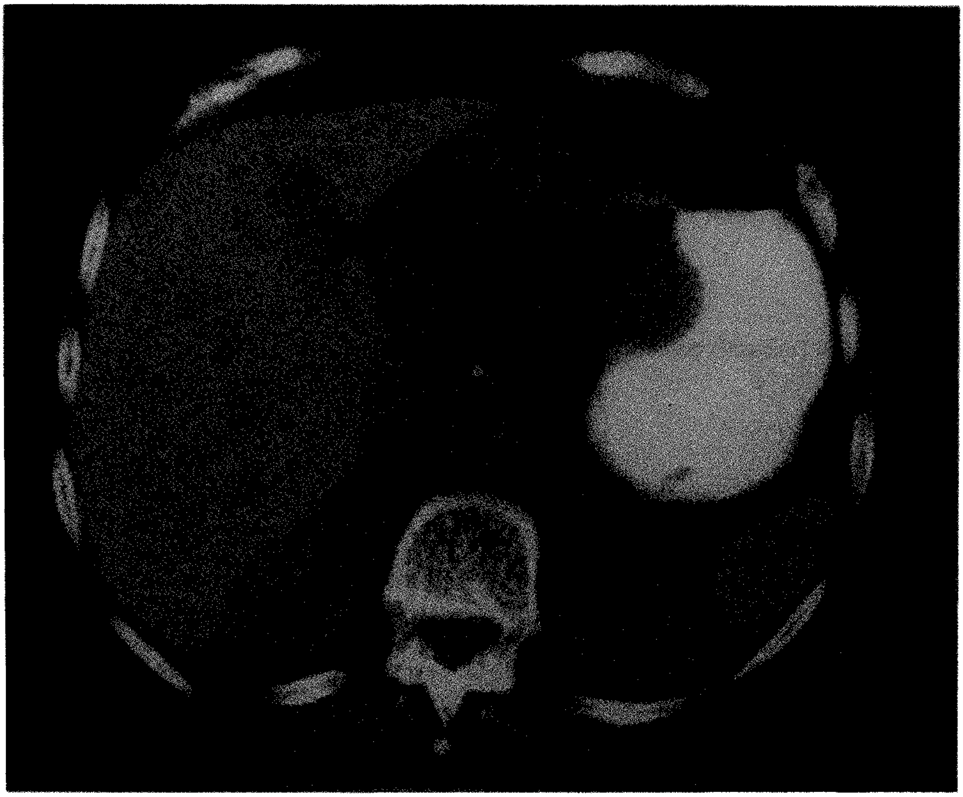

**Figure 1** CT Scan of upper abdomen showing the 8cm tumour mass in the lesser sac.

An ultrasound-guided needle biopsy showed small cystic spaces linked by cells with bland nuclei and clear cytoplasm. The cells contained much glycogen but no mucin, and immunohistochemical staining confirmed their epithelial nature. A confident diagnosis of microcystic adenoma of pancreas was made. Because of the patient's age and also because the mass has remained unchanged clinically over a period of five months and appears to be causing no symptoms, no further treatment has been given.

*Case No: 2*

A 78-year-old man was investigated for chronic constipation and vague abdominal pain radiating to the back. He had ischaemic heart disease, hypertension, Parkinson's disease, depression and chronic obstructive airways disease. He had undergone two previous transurethral resections of the prostate.

Abdominal examination showed a 3cm smooth non-tender liver edge, but was otherwise unremarkable. An ultrasound scan revealed a 7cm multicystic mass in the body of the pancreas, which was confirmed by CT scan (Figure 2). On ERCP the tumour appeared to be obstructing the main pancreatic duct in the tail of the gland. After careful consideration a decision was made to proceed to operation in this high-risk patient. A 7 cm multicystic tumour was found in the proximal body of pancreas with a dilated duct in the tail. A 50% distal pancreatectomy with splenectomy was carried out.

Histopathological examination of the specimen revealed a serous cystadenoma of the pancreas. The patient had an uncomplicated postoperative course and remains well six months later.

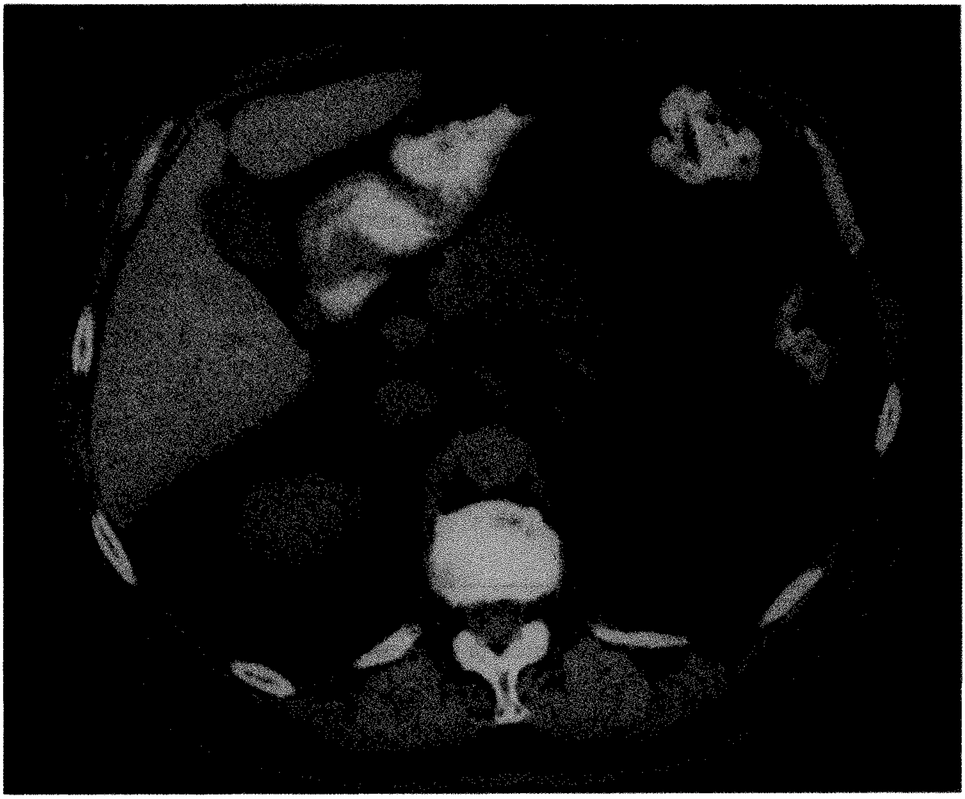

**Figure 2** CT Scan showing a multicystic pancreatic mass.

### DISCUSSION

Microcystic adenoma (serous cystadenoma) of the pancreas is a rare benign lesion, accounting for only 1% of all pancreatic tumours and 10–30% of all pancreatic cystic neoplasms[1]. It typically arises from the hed of pancreas in elderly women, and used to be regarded as a neoplasm of ductal epithelium[7], but an origin from centroacinar cells has recently been postulated[6]. Mucin secretion is either absent or very limited, and the watery secretion emanates from a simple cuboidal epithelium that is rich in glycogen[5]. Microcystic adenomas are slow-growing multilocular tumours that do not share the high malignant potential of mucinous cystic tumours, although one case of serous cystadenocarcinoma has been reported[8].

Microcystic adenomas may become large enough to compress adjacent structures sometimes leading to gastrointestinal haemorrhage or obstruction of the common bile duct or pancreatic duct[5,6]. The radiological features on CT and ultrasound scan depend on the size of the cysts and the amount of connective tissue. Loculations are a reliable criterion for a neoplastic cyst. Microcystic adenomas may be identified on CT scan (with intravenous contrast enhancement) by their multiple small (<2cm diameter) honeycomb-like loculations (seen in 50%), central scar (11%) and sunburst calcification (63%)[1,9].

The tumour is usually solitary and limited to the pancreas, although multiple lesions have been reported in patients with von Hippel-Lindau syndrome[6]. Hypervascularity on angiography is said to be a characteristic feature, but it is only present in 40% of tumours and is also seen in 33% of mucinous adenocarcinomas[1]. The combination of cytological examination with carcinoembryonic antigen and amylase assays of cyst contents may be helpful[10]. The role of MRI is under investigation, but ERCP provides little additional information[4].

The distinction between microcystic (serous) and macrocystic (mucinous) adenoma may be difficult at operation. Thus both should be excised completely whenever possible, although a frozen section biopsy may permit the surgeon to avoid unnecessarily hazardous resections of microcystic tumours. Some authors suggest that asymptomatic microcystic adenomas may be observed safely, especially in elderly and high-risk patients[5,7]. In such cases, a reliable histopathological diagnosis is essential, and percutaneous biopsy has the potential to achieve this diagnosis non-operatively. In one of our patients, the precise diagnosis was made by ultrasound-guided needle biopsy, and this allowed operation to be

**Table 1** Cystic neoplasms of the pancreas.

1. Microcystic adenoma (serous cystadenoma or glycogen rich cystadenoma)
2. Macrocystic adenoma (mucinous cystadenoma or mucinous cystic neoplasm)
3. Mucinous cystadenocarcinoma
4. Papillary cystic epithelial neoplasm (solid and papillary tumour)
5. Cystic endocrine neoplasm (cystic islet cell tumour)
6. Cystic degeneration of pancreatic adenocarcinoma (very rare)

avoided in an elderly patient with minimal symptoms. We have found only one other case of microcystic adenoma diagnosed by percutaneous biopsy[10].

Perhaps operation could have been avoided in the second patient also, though this tumour was causing some pain.

The choice of operation depends on the location of the tumour and its relationship to adjacent structures. Internal drainage and marsupialization should be avoided. Complete resection prevents the chance of recurrence from remnants of secretory epithelium, and its long-term results are excellent[4].

### *References*

1. Warshaw, A., Compton, C., Lewandrowski, K., Cardenosa, G. and Mueller, P. (1990) Cystic tumours of the pancreas. New clinical, radiologic and pathologic observations in 67 patients. *Ann. Surg.*, **212**, 432–445
2. Heatley, M., McCrory, D. and O'Hara, M. (1991) Case report: Microcystic adenoma of the pancreas. *Ulster Med. J.*, **60**, 111–113
3. von Segesser, L. and Rohner, A. (1984) Pancreatic cystadenoma and cystadenocarcinoma. *Br. J. Surg.*, **71**, 449–451
4. Hoover, E., Natesha, R., Dao, A., Adams, C. and Barnwell, S. (1991) Proliferative pancreatic cysts: pathogenesis and treatment options. *Am. J. Surg.*, **162**, 274–277
5. Compango, J. and Oertel, J. (1978) Microcystic adenomas of the pancreas (glycogen-rich cystadenomas). A clinicopathologic study of 34 cases. *Am. J. Clin. Pathol.*, **69**, 289–298
6. Alpert, L., Truong, L., Bossart, M. and Spjut, H. (1988) Microcystic adenoma (serous cystadenoma) of the pancreas. A study of 14 cases with immunohistopancreas. A study of 14 cases with immunohistochemical and electron-microscopic correlation. *Am. J. Surg. Path.*, **12**, 251--263
7. Shorten, S., Hart, W. and Petras, R. (1986) Microcystic adenomas (serous cystadenomas) of pancreas. A clinicopathologic investigation of eight cases with immunohistochemical and ultrastructural studies. *Am. J. Surg. Path.*, **10**, 365–372
8. George, D., Murphy, F., Michalski, R. and Ulmer, B. (1989) Serous cystadenocarcinoma of the pancreas: a new entity? *Am. J. Surg. Path.*, **13**, 61–66
9. Padovani, B., Neuvent, P., Chanalet, S. *et al.* (1991) Microcystic adenoma of the pancreas: report on four cases and review of the literature. *Gastrointest. Radiol.*, **16**, 62–66
10. Pinto, M. and Meriano, F. (1991) Diagnosis of cystic pancreatic lesions by cytologic examination and carcinoembryonic antigen and amylase assays of cyst contents. *Acta Cytologica*, **35**, 456–463.

*(Accepted by S. Bengmark 10 September 1992)*

